# Periodic Structural Defects in Graphene Sheets Engineered via Electron Irradiation

**DOI:** 10.3390/mi13101666

**Published:** 2022-10-03

**Authors:** Nicola Melchioni, Filippo Fabbri, Alessandro Tredicucci, Federica Bianco

**Affiliations:** 1 NEST Laboratory, Istituto Nanoscienze-CNR and Scuola Normale Superiore, Piazza San Silvestro 12, I-56127 Pisa, Italy; 2 Istituto Nanoscienze-CNR, Piazza San Silvestro 12, I-56127 Pisa, Italy; 3 Dipartimento di Fisica “E. Fermi”, Università di Pisa, Largo Bruno Pontecorvo 3, I-56127 Pisa, Italy

**Keywords:** graphene, defect engineering, low-energy electron irradiation, substrate effects

## Abstract

Artificially-induced defects in the lattice of graphene are a powerful tool for engineering the properties of the crystal, especially if organized in highly-ordered structures such as periodic arrays. A method to deterministically induce defects in graphene is to irradiate the crystal with low-energy (<20 keV) electrons delivered by a scanning electron microscope. However, the nanometric precision granted by the focused beam can be hindered by the pattern irradiation itself due to the small lateral separation among the elements, which can prevent the generation of sharp features. An accurate analysis of the achievable resolution is thus essential for practical applications. To this end, we investigated patterns generated by low-energy electron irradiation combining atomic force microscopy and micro-Raman spectroscopy measurements. We proved that it is possible to create well-defined periodic patterns with precision of a few tens of nanometers. We found that the defected lines are influenced by electrons back-scattered by the substrate, which limit the achievable resolution. We provided a model that takes into account such substrate effects. The findings of our study allow the design and easily accessible fabrication of graphene devices featuring complex defect engineering, with a remarkable impact on technologies exploiting the increased surface reactivity.

## 1. Introduction

Defects in the crystal lattice of graphene are usually undesired as they hinder many of the remarkable properties of graphene devices. For instance, the presence of defects causes the broadening of the phonon dispersion and the lowering of the electronic mobility [1,2]. However, structural defects can also have a beneficial impact, especially when designing graphene devices with specific characteristics for particular applications. Indeed, by deterministically inducing defects in the lattice, it is possible to engineer the chemical, thermal, electronic, and mechanical properties of graphene and to conceive devices with novel functionalities [3,4]. As an example, the presence of defects enhances the surface chemical reactivity of graphene [5,6,7], with a great impact on the design of devices that exploit the altered chemical functionalization for applications in sensing, energy harvesting, and energy storage [8,9]. Patterning predetermined defective structures on the graphene surface would also largely contribute to solid-state quantum technologies. In fact, the energy and charge transport properties can be tuned by defect engineering [6]. In general, the presence of defects can induce strong localization of electrons in the crystal [6] and reduces the electronic mobility [2], thus allowing the fabrication of complex conductive/insulating structures. Structural defects also reduce the thermal phonon conductivity [10], causing an increase in the thermoelectricity (up to threefold) at room temperature [11], and find applications in electronic devices such as radiation sensors [12].

When periodically repeating defected features, additional novel graphene functionalities can be unlocked. For example, a periodic array of defect lines in a graphene sheet can further tailor graphene’s thermal properties by introducing an anisotropy in the thermal conductivity [13,14]. Multiple line defects have been also predicted to enhance the control of the valley degree of freedom in valleytronics devices [15] or to generate superlattices that enable the fine tuning of the band structure [16]. Finally, mastering the deterministic generation of defects in graphene can also represent an additional aspect of studying the light–matter interaction. Indeed, artificially induced lines of defects have been demonstrated to be effective reflective boundaries for plasmons [17].

Low-energy (<20 keV, [18]) electron beam irradiation (EBI) stands out as a valuable technique for modifying the graphene lattice and achieving full control over engineered defects. Even if the knock-on threshold of carbon atoms in graphene is much higher than the energy transferred by a single collision of a low-energy electron [19], structural defects are nonetheless expected to form when considering alternative physical mechanisms. One of the possible mechanisms relies on the fact that the impact resistance of graphene is reduced when the lattice already contains some defects and is supported by a substrate [20]. Alternatively, lattice disruption is expected due to the accumulation of charged puddles on the surface or within the subsurface [21]. Such puddles create electrostatic fields that can reach instant values that are sufficiently strong to induce severe damage to the graphene lattice [21,22]. Moreover, beam-induced chemical etching can be a viable mechanism for the creation of defects. The chemical species that might be responsible for such effects can be reactive oxygen-based compounds coming from the substrate [23] or from organic residues trapped between the graphene and the substrate [24]. Considering these effects, defective patterns with nanometric precision can be achieved using a pattern generator-equipped scanning electron microscope (SEM) [3,6,25,26].

Defect-engineered graphene for plasmonic and electronic applications requires well-defined patterns, where defect-rich and defect-free areas are well distinguished. For this reason, when introducing defective patterns such as line gratings via EBI, the investigation of both the defect density and surface topography is fundamental in order to establish the efficacy of the pattern creation. In this context, we present the study of the periodic arrays of defective lines induced in exfoliated graphene on a silicon oxide/silicon (${\mathrm{SiO}}_{2}/\mathrm{Si}$) substrate by irradiating with an electron beam (e-beam) at 20 keV. We investigate both the morphological modifications of the graphene sheet due to EBI by atomic force microscopy (AFM) measurements and quantify the induced defects by micro-Raman spectroscopy when varying the distance among the defective lines. Interestingly, both the topography and the two-dimensional (2D) defect density exhibit a notable dependency on the pattern pitch *p*, showing that the resolution of a multiple-line pattern is not only determined by the EBI system characteristics (e.g. e-beam energy, spot size, etc.) but is also limited by the pattern parameters themselves.

## 2. Materials and Methods

Monolayer graphene flakes were deposited on n-type Si substrates with 300 nm of thermally-grown ${\mathrm{SiO}}_{2}$ on top by the micro-mechanical exfoliation of highly oriented pyrolytic graphite. Before the graphene deposition, the substrate chips were cleaned by oxygen plasma at 100 W for 5 minutes to promote the adhesion of graphene and remove organic residues on the surface as they affect the defect patterning definition [26].

**Figure 1:**
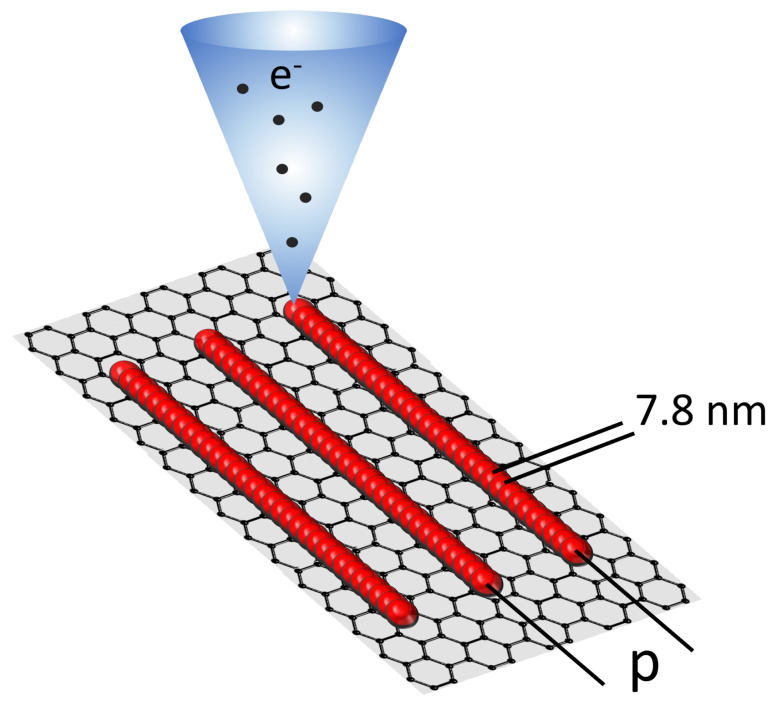
Sketch of the electron irradiation: red spots indicate the landing points of the primary e-beam. All lines are exposed with a step size of 7.8 nm (distance between the landing points) and are separated by a distance *p*.

Defects were induced only in some areas of the graphene surface by irradiating with electrons accelerated at 20 keV and creating arrays of lines with different geometries, as depicted in Figure 1. The pattern pitch *p* varied from 20 nm to 100 nm. The step-size along the lines was set at 7.8 nm. For each pattern, the e-beam current was about 100 pA and delivered a dose of ∼31 mC/cm, resulting in a dwell time of ∼250 μs. Two devices were fabricated and are referred to as “Set 1” and “Set 2” in the text. Both devices were fabricated using identical steps to test the reproducibility of the experiment.

The morphology of the patterns was studied by AFM. The flakes were scanned with the microscope set to tapping mode to reduce the possible interaction between the tip and the sample. The AFM topographic maps were acquired in high-resolution mode (1024 × 1024 pixels) with a scanning velocity of 500 nm/min. Such settings allowed a resolution of ∼2 nm/px.

The impact of the defects on the crystal lattice was studied by micro-Raman spectroscopy just after the electron irradiation in ambient air. The flakes were analysed by a 532 nm laser with 100× objective (NA = 0.85), giving a lateral resolution <1 μm. The laser power was set at 118 μW to exclude any possible laser heating of the lattice, which can cause partial healing of the defects. Map scans were taken on all the flakes so that information on both the defected and non-irradiated graphene was accessible using the same measurements. Since the micro-Raman did not have sufficient spatial resolution to distinguish between the lines of the created patterns, all the data extracted from the Raman spectra were averaged over each pattern area. The Raman signal collected on the pristine part of the graphene flake was used as a reference.

## 3. Results and Discussion

AFM and micro-Raman were performed to study the role of the pattern pitch on the surface topography and the defect density of electron-irradiated graphene sheets.

**Figure 2:**
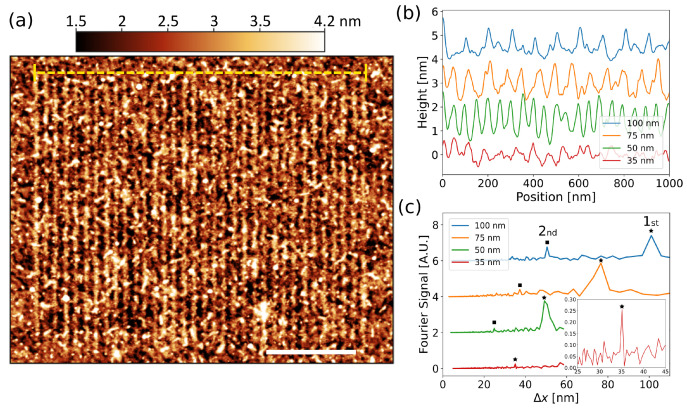
(**a**) Example of topographic height map for Set 1 irradiated with a 50 nm pattern. The patterned area is highlighted by the yellow dashed line. The scale bar is 500 nm. (**b**) Comparison of lines extracted from AFM scans of patterns with different pitches, as reported in the legend. Each line average is shifted 1.5 nm. (**c**) Fast Fourier transform of the lines reported in (**b**). First harmonic peaks are indicated as “1st” and by stars, second harmonic peaks as “2nd” and by squares. Inset is a zoomed-in image of the FFT of the height profile for *p* = 35 nm.

In the AFM scans, the patterned arrays always resulted in a regular local increase of the topographic height. An example of an AFM map is shown in Figure 2a, where the local height variations induced by the defective line-grating pattern with a pitch of 50 nm (Set 1) are clearly distinguishable (see also Figure S1 for an AFM overview of the different patterns in Supplementary Information). The increase in the topographic height may be attributed to the presence of contaminants that were adsorbed by part of the defected sites due to the air exposure [18,27]. Instead, no height modifications were measured on the pristine areas of the graphene flake and when irradiating the substrate, even when exposed for longer times (see Figure S2). The observations on the substrate excluded that the height increase was due to the possible deposition of amorphous carbon during the EBI.

**Figure 3:**
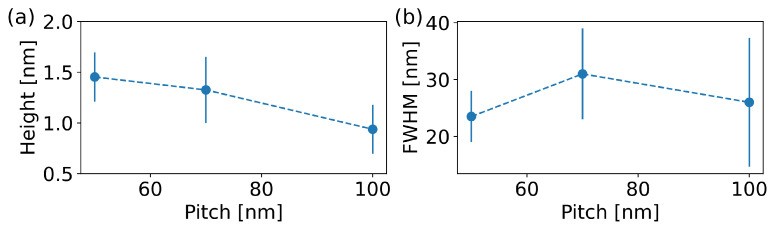
(**a**) Average height of the peaks measured via AFM as a function of the pitch *p*. (**b**) Average FWHM of the peaks measured via AFM as a function of the pitch *p*. Standard deviations are used as error bars.

To analyse the height variations, a single profile line was extracted from the AFM scans by averaging multiple lines for all the patterns. As shown in Figure 2b, clear periodic oscillations are discernible for pitch *p* of as low as 50 nm. Contrarily, the extracted line for 35 nm shows an overall height increase of ∼1 nm with respect to unperturbed graphene (see Figure S1), albeit no regular pattern can be easily recognized. To better identify the periodicity of the observed height oscillations, we performed a fast Fourier transform (FFT) on the height profiles measured for each pattern. Figure 2c shows that the Fourier signals for *p* varying from 50 nm to 100 nm exhibit a clear first harmonic peak at Δx=p and also a second harmonic peak at Δx=p/2, demonstrating that the array was ordered. Interestingly, the FFT of the pattern with *p* = 35 nm shows a distinct first harmonic peak at 35 nm (see inset in Figure 2c), indicating that a regular defective pattern was created even though the height profile did not show discernible order. This behaviour can be explained by considering that the focused electron beam had a finite radius $r_{B}$ when impinging on the chip. In the present study, such a radius is of the order of tens of nm. Consequently, when the pitch *p* approached the dimension of the radius, the quality of the pattern was expected to decrease in terms of lateral resolution. However, when fitting all profiles with arrays of Lorentzian peaks to extract the height and full width half maximum (FWHM) of the height oscillations, we observed a pitch dependency of the defective graphene topography also for the patterns having a pitch longer than the beam radius. In particular, as shown in Figure 3a, the height of the oscillation peaks decreased as *p* increased, suggesting an unexpected dependency of the surface chemical reactivity on the pattern pitch and thus a variation of the defect density when changing *p*. Instead, the width of the patterned lines was constant within the error at a value that is compatible with the e-beam spot dimensions (Figure 3b), demonstrating that the lines’ lateral resolution was not detrimentally affected by the patterned irradiation. A possible explanation of the *p* dependency of the height may arise from the interaction of electrons back-scattered by the substrate (BSEs) with the adjacent lines previously irradiated. Indeed, BSEs retain most of the energy of the primary electrons and can be spread in an area of radius $r_{BSE}>r_{B}$, or up to a few micrometers around the irradiated spot [26]. Therefore, along the lines, the defects were mostly generated by the interplay of both primary and back-scattered electrons. Instead, in between the lines, only BSEs caused lattice modifications. BSEs are less numerous compared to primary electrons and their kinetic energy varies with the radial distance from the primary beam-landing position. Thus, the quantity of defects induced by BSEs was expected to be lower than that induced by primary electrons [26]. Indeed, we observed an increase in the height within the lines but no inter-diffusion of adsorbates between adjacent lines (constant peak FWHM), indicating that the surface reactivity was enhanced primarily in the pattern lines, where the defect density was the highest [28].

**Figure 4:**
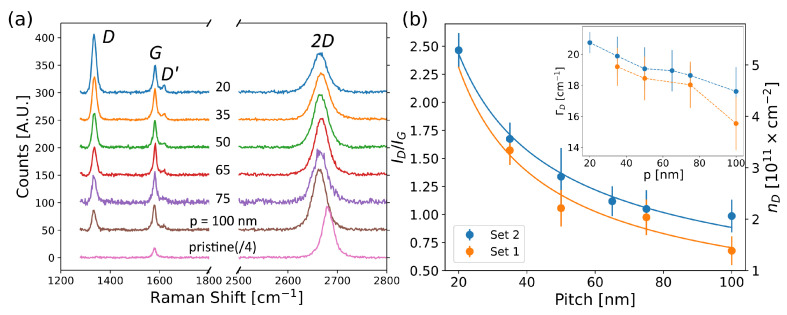
(**a**) Averaged Raman spectra taken on graphene patterned with different pitches *p*. Each spectrum is shifted 50 A.U. and the spectrum collected on the pristine graphene is divided by 4. (**b**) Plot of the ratio $I_{D}/I_{G}$ of the intensities of the *D* and *G* peaks as a function of the pitch *p* of the pattern. Full lines are the fitting curves based on Equation (2). Inset, the width $\Gamma_{D}$ of the *D* peak is plotted as a function of the pitch *p*. Standard deviations are used as error bars.

From the lattice point of view, quantitative information on the electron-induced defects were extracted via micro-Raman spectroscopy. The averaged Raman spectra of each pattern and pristine graphene are reported in Figure 4a. As expected, we observed the appearance of the defect-activated Raman bands (*D* and $D^{\prime }$ peaks) [29,30] when creating the defective patterns. The presence of defects was also confirmed by the quenched 2D peak with respect to the one measured on pristine graphene [31] and their natures varied from a majority of vacancy/boundary-like defects for long pitches to the coexistence of sp^3^-like and vacancy/boundary-like defects reported for short pitches (see Section S2 in Supplementary Information). The peak intensity of defect-activated Raman bands ($I_{D}$, $I_{D^{\prime }}$) increased when the pitch of the pattern decreased. This is translated in a *D* over *G* peak-intensity ratio ($I_{D}/I_{G}$) that increased when reducing the pattern pitch, as shown in Figure 4b for both Set 1 and Set 2. The corresponding 2D densities of the defects ($n_{D}$) were computed using the formula reported in [32]:

(1) $$n_{D}\left[{\mathrm{cm}}^{-2}\right]=7.3\cdot 10^{9}E_{L}^{4}\left[{\mathrm{eV}}^{4}\right]\frac{I_{D}}{I_{G}},$$

where $E_{L}$ is the energy of the employed Raman laser expressed in eV (see Section 2). The extracted densities varied from ∼2 $\times 10^{11}$ cm^−2^ at *p* = 100 nm to $5.26\times 10^{11}$ cm^−2^ at *p* = 20 nm. All $I_{D}/I_{G}$ maps and relative optical images of Set 1 and Set 2 are reported in Figures S3 and S4. An increase in the density of the defects when reducing *p* was also confirmed by the analysis of the width of the *D* peak ($\Gamma_{D}$). Typically, an enlarging of $\Gamma_{D}$ is associated with an increase in the density of the defects [33,34]. As shown in the inset in Figure 4b, in Set 1 and Set 2 the *D* peak monotonically broadened when reducing the pitch *p*, demonstrating that more defects were created at shorter pitches.

To interpret the observed *p* dependency of the induced defects, we have to consider that the EBI induces a certain density of defects $\rho_{sl}$ along a single line. In each pattern, the density of lines (i.e., lines per unit length) is the inverse pitch $n_{L}=1/p$. Therefore, the density of defects induced in a graphene sheet can be expressed as $n_{D}=\rho_{sl}\cdot n_{L}=\rho_{sl}/p$. The linear density $\rho_{sl}$ is expected to be identical in all lines and patterns as the same irradiation parameters are used. However, the amount of defects induced along a single line is influenced by BSEs and is not independent on the pattern pitch, as also suggested by the AFM results. Thus, we fit the experimental data $I_{D}/I_{G}$ with the following function:

(2) $$\frac{I_{D}}{I_{G}}\left(p\right)=\frac{A}{p}\cdot {\left(\frac{p}{r_{BSE}}\right)}^{B},$$

where $r_{BSE}=1.5\;\mu m$ is the radius of BSE for 20 keV irradiation [26] and *A* has the same unit of *p*. The fitting parameter values are listed in Table 1 for both sets of samples. Here, we assumed a slow power-law dependence of $\rho_{sl}$ with the pitch $\rho_{sl}\propto p^{B}$ with 0<B<1. The obtained $I_{D}/I_{G}$ ratio can be converted in terms of $n_{D}$ with Equation (1). Defining α as the value of *A* transposed by the equation, the term $\alpha {\left(p/r_{BSE}\right)}^{B}$ can be interpreted as the effective induced linear density of the defects $\rho_{sl}^{eff}$, which takes into account the effect of all graphene-interacting electrons, coming from both the primary beam and back-scattering events.

**Figure 5:**
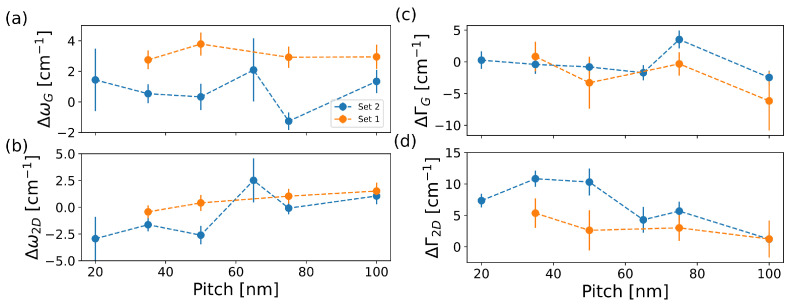
(**a**,**b**) Changes in the *G* and 2D peak positions with respect to the pristine flake as a function of the pitch *p*. Here, $\Delta \omega_{G,2D}=\omega_{G,2D}^{def}-\omega_{G,2D}^{0}$, where $\omega_{G,2D}^{0}$ and $\omega_{G,2D}^{def}$ are the positions of the *G* and 2D peaks before and after the irradiation, respectively. (**c**,**d**) Changes in the *G* and 2D peak widths with respect to the pristine flake as a function of the pitch *p*. Here, $\Delta \Gamma_{G,2D}=\Gamma_{G,2D}^{def}-\Gamma_{G,2D}^{0}$, where $\Gamma_{G,2D}^{0}$ and $\Gamma_{G,2D}^{def}$ are the widths of the *G* and 2D peaks before and after the irradiation, respectively. Standard deviations are used as error bars.

To investigate how the pattern pitch influenced the doping and strain of the defective graphene flakes, we studied the behaviour of the positions of the *G* ($\omega_{G}$) and 2D ($\omega_{2D}$) peaks. Figure 5a,b report the plot of the shift of the *G* and 2D peak positions with respect to their values in pristine graphene ($\Delta \omega_{G,2D}=\omega_{G,2D}^{def}-\omega_{G,2D}^{0}$) for the various pitches in Set 1 and Set 2, respectively. First, we observed that the electronic irradiation moderately blue shifted the *G* peaks, which means that there was a change in the doping of the crystal, i.e., from ∼3–4 $\cdot 10^{12}\;{\mathrm{cm}}^{-2}$ in pristine graphene to ∼5–6 $\cdot 10^{12}\;{\mathrm{cm}}^{-2}$ in irradiated graphene. In addition, there was a weak dependence of the doping on *p*, which went from ∼4.5 $\cdot 10^{12}\;{\mathrm{cm}}^{-2}$ for *p* = 100 nm to ∼6.5 $\cdot 10^{12}\;{\mathrm{cm}}^{-2}$ for *p* = 20 nm [35] This behaviour was confirmed by the slightly detectable change in the difference $\Delta \;\Gamma_{G}$ ($\Delta \Gamma_{G,2D}\;=\;\Gamma_{G,2D}^{def}\;-\;\Gamma_{G,2D}^{0}$) of the widths of the *G* peak before and after the irradiation [33], as reported in Figure 5c. In electron-irradiated graphene, the width of the *G* peak is determined by the competitive action of the carrier concentration, which narrows the *G* peak and defects density, which broadens the peak. However, electron-defect scattering mainly dominated when $I_{D}/I_{G}$ ≥ 3 [34] and in both sets, $I_{D}/I_{G}$ was always lower than 3. In general, this local irradiation-induced increase in the charge carrier density may be attributed to the surface activation (and thus to the adsorbants) and/or to the interaction with the substrate [36]. Indeed, during electron exposure, charge carries can be created in the substrate. These charges can last up to ∼10 h after the irradiation [36,37], thus altering the net defect-related doping in the crystal.

On the other hand, $\omega_{2D}$ was red shifted and $\Delta \Gamma_{2D}$ decreased as the pitch increased (see Figure 5b,d). The 2D peak behaviour was an indication of an increase in the graphene tensile strain when reducing the pattern pitch [38]. Indeed, $\omega_{2D}$ was almost independent of defect density until $I_{D}/I_{G}$ ≤ 3 [34,39], whereas the 2D peak broadened when increasing the defect density and/or the overall tensile strain [31,40]. As a result, in Set 1, the graphene strain was quantified, varying from +0.31% for *p* = 100 nm to +0.38% at *p* = 20 nm. In Set 2, it varied from +0.2% for *p* = 100 nm to +0.3% at *p* = 35 nm [35].

## 4. Conclusions

The features of periodic arrays of structural defects in graphene were studied by combining AFM and micro-Raman spectroscopy. Defected lines were generated by low-energy EBI in monolayer graphene. The AFM measurements demonstrated that it was possible to create well-defined periodic patterns with a pitch *p* as small as 50nm. For lower resolutions, the effect of EBI was still visible, but the characteristic length approached the radius of the beam $r_{B}$, with detrimental effects on the long-range order of the array. By analysing the patterns with pitch p≥50 nm, we found that the average width of the defective lines was comparable to the e-beam spot size, thus confirming the quality of the generated arrays.

The analysis of the Raman signal showed that the 2D density of defects induced in the crystal increased when the distance between each line decreased. Both Raman and AFM analyses confirmed that such a change was not a simple 1/p dependency expected due to the increasing density of the lines (i.e., number of defects in the measured spot), but rather because the substrate had an impact on the quality and the achievable resolution of the patterned arrays. In fact, the substrate was responsible for the back-scattering of the primary electrons, which can interact with the previously defected lattice. Indeed, by taking into account the effect of BSEs, we built a model to extract the induced effective linear density of defects $\rho_{sl}^{eff}$. Such a presented model allowed for the fine tuning of the irradiation parameters to push the resolution as high as possible when fabricating real devices.

In conclusion, we demonstrated that it was possible to generate periodic arrays of defects in a graphene sheet, which could be exploited for designing engineered electronic devices or for inducing optically active features of unprecedented resolutions. Such features, combined with the increased surface reactivity of defected graphene, could have a great impact on both novel solid-state studies and the design of closely-packed chemical and radiation sensors.

## Figures and Tables

**Table 1:** Fit results for the data reported in Figure 1b in the main text. α is the value of *A* transposed in the density of defects by Equation (1).

Set	A [nm]	B	$\alpha \;[10^{11}{\mathrm{cm}}^{-2}\cdot \mathrm{nm}$]
1	241	0.37	520
2	141	0.26	307

## Data Availability

All the data are available upon request to the corresponding author.

## Supplementary Materials

The following supporting information can be downloaded at: https://www.mdpi.com/article/10.3390/mi13101666/s1, Figure S1: AFM scans of the patterned arrays of Set 1; Figure S2: AFM scan and cut lines of the patterned arrays on graphene and substrate for comparison; Figure S3: $I_{D}/I_{D^{\prime }}$ ratio as a function of the pitch for the data of Set 1; Figure S4: $I_{D}/I_{G}$ spatial distribution for all patterns of Set 1; Figure S5: $I_{D}/I_{G}$ spatial distribution for all patterns of Set 2.

## Abbreviations

The following abbreviations are used in this manuscript:

EBI	Electron Beam Irradiation
SEM	Scanning Electron Microscopy
AFM	Atomic Force Microscopy
BSE	Back-Scattered Electron
FFT	Fast Fourier Transform
FWHM	Full Width at Half Maximum
