# Supplementary material for: Periodic Structural Defects in Graphene Sheets Engineered via Electron Irradiation
Source: Micromachines (Basel). 2022 Oct 3;13(10):1666. doi: 10.3390/mi13101666 (PMC9606931; doi:10.3390/mi13101666)
Supplement: Supplementary file 1 [file micromachines-13-01666-s001.zip › micromachines-1928019 - supplementary.pdf]

Periodic structural defects in graphene sheets engineered via electron-irradiation
Supplementary Material

Nicola Melchioni[1], Filippo Fabbri[1], Alessandro Tredicucci[2,3], and Federica Bianco[1,*]

[1]*NEST, Istituto Nanoscienze-CNR and Scuola Normale Superiore, Piazza San Silvestro 12, 56127, Pisa*
[2]*Istituto Nanoscienze-CNR Piazza San Silvestro 12, I-56127, Pisa, Italy*
[3]*Dipartimento di Fisica "E. Fermi", Università di Pisa, Largo B. Pontecorvo 3, I-56127, Pisa*
[*]*Corresponding author: federica.bianco@nano.cnr.it*

S1 Topographic height mapping for all patterns of Set 1

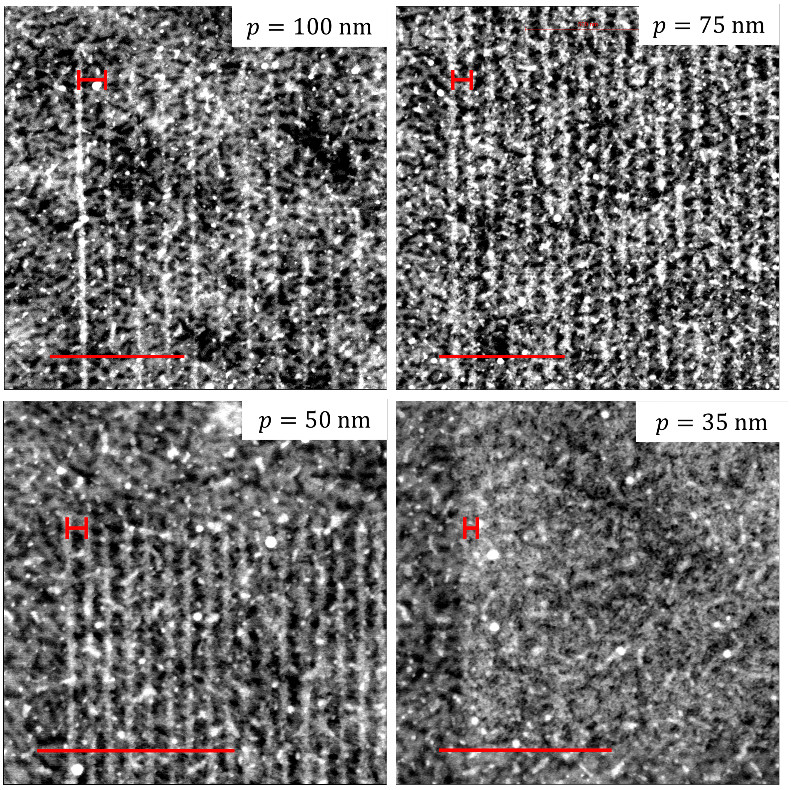

Figure S1: AFM scans of the patterned arrays of Set 1. The scale bars is 500 nm. The nominal pitch of the corresponding pattern is reported and over-imposed to two adjacent lines.

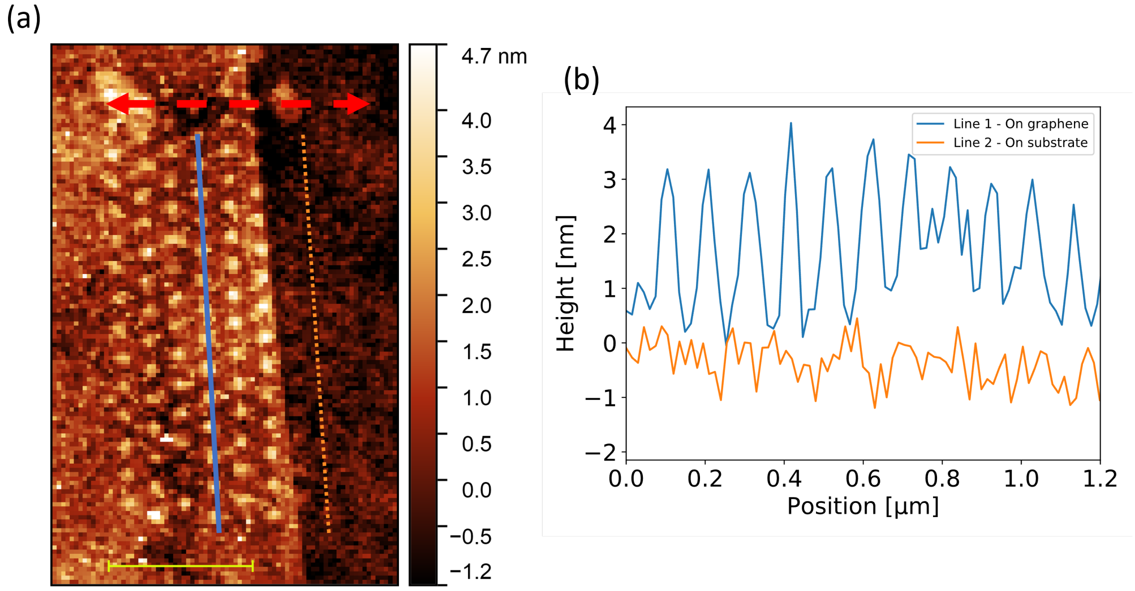

Figure S2: (a) AFM scans of a patterned area on both graphene (left) and substrate (right). The scale bars is 500 nm. The red dashed double-arrow indicates the exposed area. The blue solid line and the orange dotted line are cut lines plotted in (b). Such single points exposition was obtained by setting the SEM pattern generator to deliver a current of 0.15 nA with a dose of 40 mC/cm^2, resulting in a dwell time of 30 ms, much higher than the one used for the line arrays ($\sim$ 0.25 ms). (b) Height measurement along the blue solid line (line 1) and orange dotted line (line 2) shown in (a).

S2 Analysis of the D' peak to determine the nature of the defects in Set 1

By following the method of Eckmann et al. (Ref. 30 in the main text), the ratio of the intensities $I_D/I_{D'}$ of the intensities of the D and D' peaks can be exploited to gain information on the nature of defects induced in the crystal with the irradiation. In the presented case, the ratio decreases as the pitch p increases, suggesting that the defect nature shifts from a majority of vacancy/boundary-like defects for large pitches towards a coexistence of sp^3-like and vacancy/boundary-like defects for short pitches. Indeed, the $I_D/I_{D'}$ ratio never reaches the value of 13 (i.e. the value measured when the population of defects is mostly sp^3-like). Although the pitch-dependence of the defect nature is not clearly understood and requires further studies, we can tentatively explain it as follows. When the irradiated areas are clearly distinguishable from the non-irradiated areas (i.e. for pitches $p > r_B$), the defective sites have low defects density and are rich of vacancy-like defects, as indicated by the I_D/I_G (< 1) and I_D/I_D' (~ 6) values, respectively. As the pitch is reduced, the 2D density of defects increases (see Fig. 4 in the main text). This increase in the density of defects might lead towards a more pronounced local corrugation of the lattice, also due to the interaction with the supporting substrate, as suggested by the observed increase in strain and doping for shorter pitches (see Fig. 5 and related discussion in the main text). This corrugation and the increased chemical reactivity due to the higher defect density (in this case, the presence of adatoms is more favored compared to the lower defect density) may explain the deduced population that is an average effect of the coexistence of sp^3-like and vacancy-like defects.

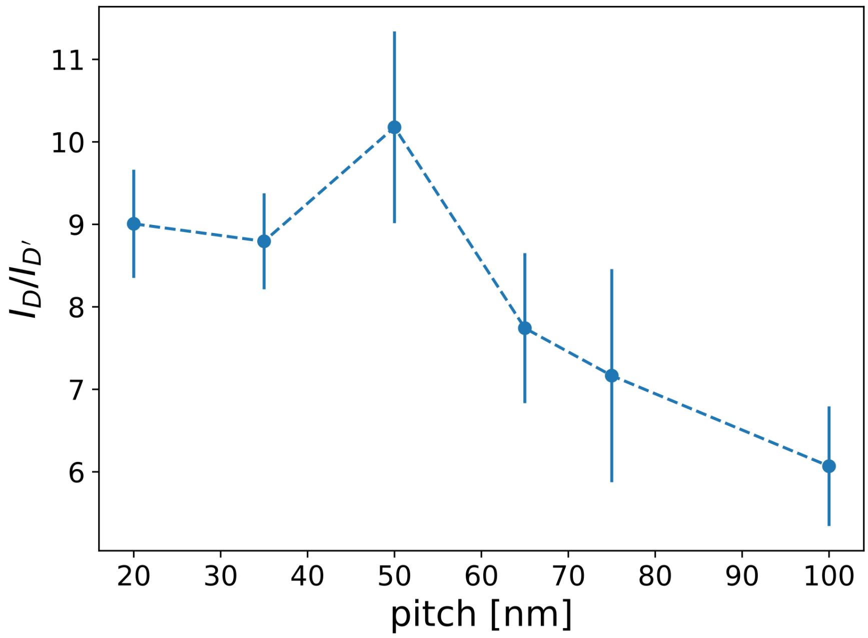

Figure S3: Plot of the ratio $I_D/I_{D'}$ of the intensities of the D and D' peaks as a function of the pitch p for Set 1.

S3 I_D/I_G spatial distribution for all patterns of Set 1 and Set 2

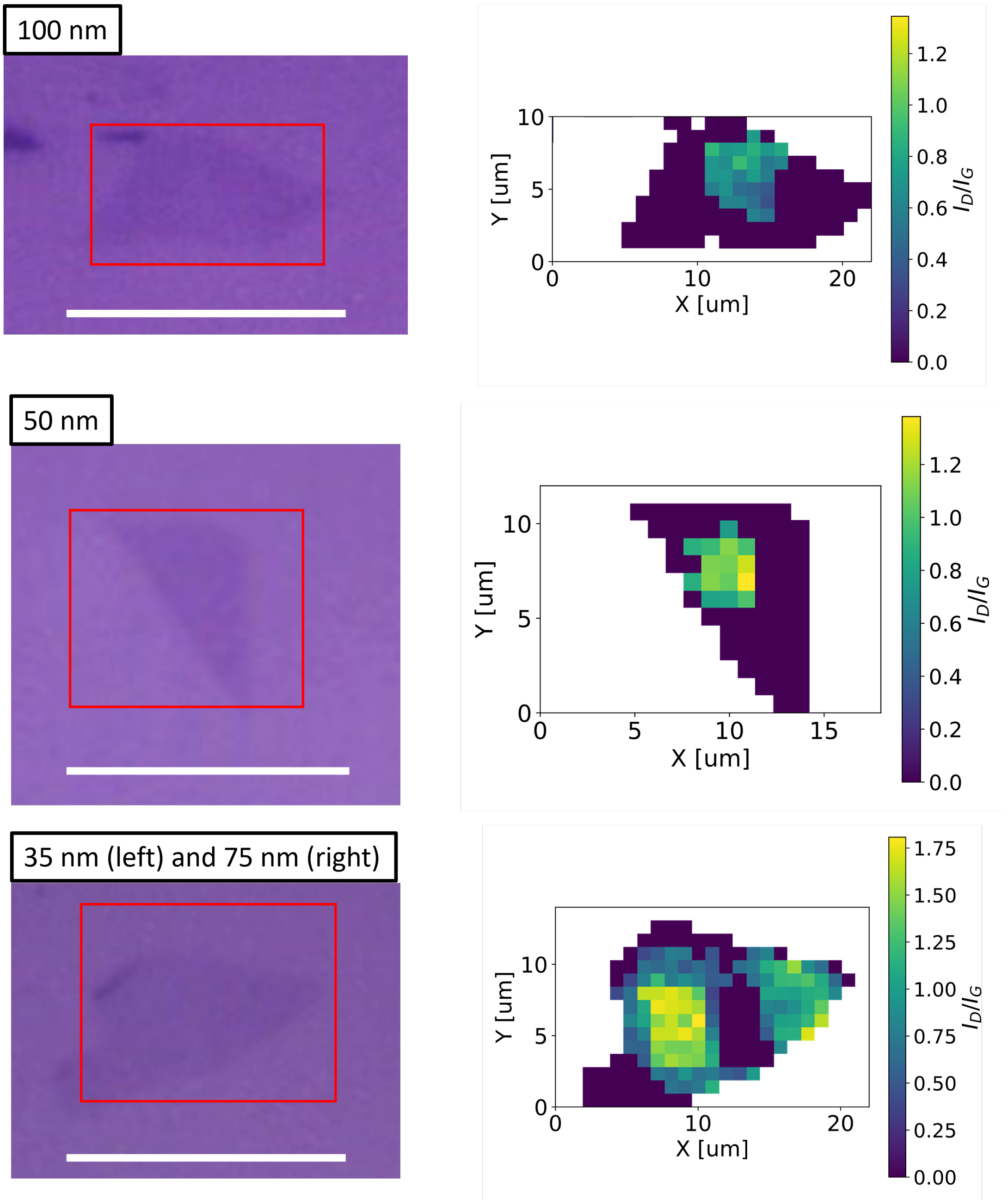

Figure S4: Optical images (left) and map of the intensity ratio of *D* and *G* peaks (right) for flakes of Set 1 in the main text. The nominal pitch of the corresponding pattern is reported above the optical images. The scale bar is 10 μm in all the optical images. The red solid lines enclose the area mapped with Raman.

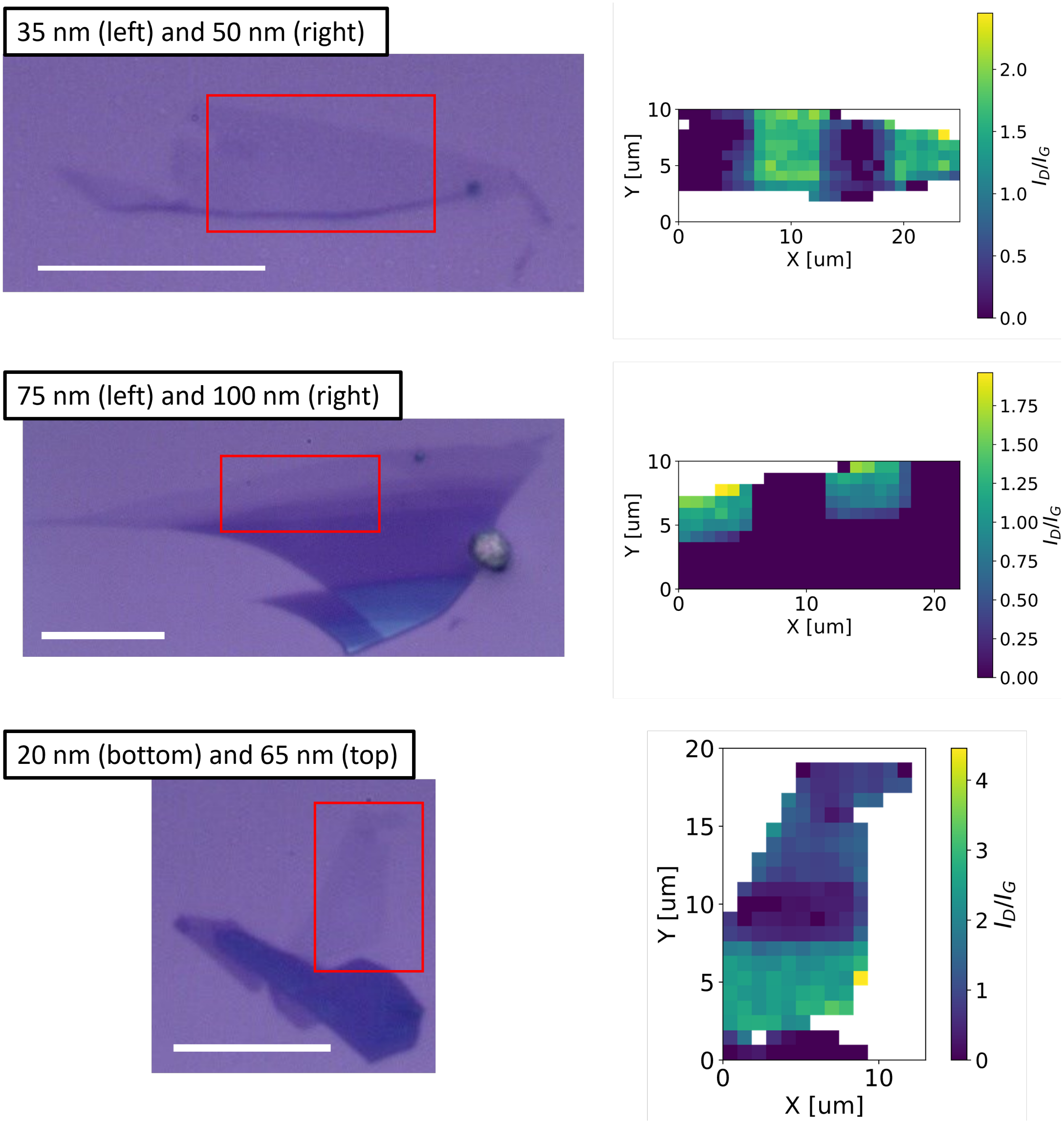

Figure S5: Optical images (left) and map of the intensity ratio of D and G peaks (right) for flakes of Set 2 in the main text. The nominal pitch of the corresponding pattern is reported above the optical images. The scale bar is 10 μm in all the optical images. The red solid lines enclose the area mapped with Raman.
